# ARE THERE BIDIRECTIONAL ASSOCIATIONS BETWEEN COGNITION AND BALANCE ABILITY: EVIDENCE FROM A BRITISH BIRTH COHORT

**DOI:** 10.1093/geroni/igz038.2268

**Published:** 2019-11-08

**Authors:** Joanna M Blodgett, Rachel Cooper, Daniel Davis, Diana Kuh, Rebecca Hardy

**Affiliations:** 1 UCL, London, United Kingdom; 2 Independent author, London, United Kingdom

## Abstract

Age-related changes in cognitive and balance abilities are well-established, as is their correlation with one another. There is, however, limited evidence regarding the directionality of associations and whether or not common biological processes may underlie their age-related declines. The main aim was to explore bidirectional associations between balance and cognitive abilities in mid-later life using data from the MRC National Survey of Health and Development, the 1946 British birth cohort (n=2735). Cognition was assessed at ages 43, 53, 60-64 and 69 with verbal memory and search speed tasks. One-legged standing balance time (eyes closed) was assessed at ages 53, 60-64 and 69. Two autoregressive cross-lagged models simultaneously assessed bidirectional associations of balance with verbal memory and search speed over time. Results suggest a unidirectional association between verbal memory and subsequent balance in both sexes, decreasing with age from 0.14 SD balance (95%CI: 0.10,0.17) per 1SD verbal memory to 0.06 (0.01,0.10) to 0.05 (0.01,0.09). Search speed at age 43 was associated with balance at age 53 [men: 0.11(0.06,0.16); women: 0.09 (0.03,0.13)]; additionally, in men, there was evidence of a bidirectional association between ages 60-64 and 69 [balance to search speed: 0.05 (0.00,0.10); search speed to balance: 0.09 (0.02,0.16)]. These findings support the notion that successful balance relies mainly upon cognitive processing to successfully integrate vestibular, visual and proprioceptive input with motor output. Including a cognitive component in balance and fall risk intervention programs could have an additive benefit in improving neural pathways involved in balance and thus reducing fall risk.

